# A206 DUPILUMAB TREATMENT REDUCES THE FREQUENCY OF DYSPHAGIA DAYS AND ACTIONS TO RELIEVE DYSPHAGIA IN PATIENTS WITH EOSINOPHILIC ESOPHAGITIS (EOE): RESULTS FROM THE PHASE 3 LIBERTY EOE TREET STUDY

**DOI:** 10.1093/jcag/gwad061.206

**Published:** 2024-02-14

**Authors:** E Dellon, I Hirano, M Chehade, N Fugere, X Sun, T Pela, S Durrani, J A Jacob-Nara, A Radwan, S T Tilton, E McCann

**Affiliations:** University of North Carolina School of Medicine, Chapel Hill, NC; Northwestern University Feinberg School of Medicine, Chicago, IL; Icahn School of Medicine at Mount Sinai, New York, NY; Sanofi, Mississauga, ON, Canada; Regeneron Pharmaceuticals Inc., Tarrytown, NY; Sanofi, Bridgewater, NJ; Regeneron Pharmaceuticals Inc., Tarrytown, NY; Sanofi, Bridgewater, NJ; Regeneron Pharmaceuticals Inc., Tarrytown, NY; Sanofi, Bridgewater, NJ; Regeneron Pharmaceuticals Inc., Tarrytown, NY

## Abstract

**Background:**

Dysphagia is the predominant symptom of EoE in adolescents and adults.

**Aims:**

To assess change in number of days with dysphagia, number of days with action taken to relieve dysphagia, and change in pain associated with dysphagia in patients receiving dupilumab 300 mg once weekly (qw) vs placebo in LIBERTY EoE TREET.

**Methods:**

Dysphagia Symptom Questionnaire (DSQ) score (range 0–84) was calculated from daily responses over 14 days for Q2 (frequency) and Q3 (severity); Q4 (pain) was separately assessed (range 0–56). Higher scores indicate a worse outcome. Inclusion criteria required patients to have a baseline DSQ score ≥10. Number of days with dysphagia (Q2), action taken to relieve dysphagia (Q3), and pain (Q4) were analyzed at baseline and Week (W) 24 for the placebo-controlled study parts (A and B). *P*-values are nominal.

**Results:**

81 patients enrolled in Part A (dupilumab/placebo: n=42/39) and 159 in Part B (n=80/79). Mean number of days with dysphagia was similar with dupilumab vs placebo at baseline, but lower in the dupilumab group at W24 (**Figure**). Mean [SD] change from baseline (dupilumab vs placebo) was –6.5 [3.78] vs –3.9 [4.61], *P*ampersand:003C0.05 in Part A, and –7.8 [4.24] vs –4.7 [4.02], *P*ampersand:003C0.0001 in Part B. Mean [SD] number of days with any action taken to relieve dysphagia was similar in the dupilumab vs placebo group at baseline (Part A: 7.3 [3.76] vs 8.2 [3.82]; Part B: 9.5 [3.53] vs 8.7 [3.85]) but lower (numerically) in the dupilumab group at W24 (Part A: 3.6 [3.73] vs 5.7 [4.78]; Part B: 3.0 [3.84] vs 5.3 [4.69]). At W24, numerically fewer days with more severe dysphagia (need to cough/gag, vomit, or seek medical attention to relieve dysphagia) occurred with dupilumab vs placebo. Baseline DSQ pain score was similar in dupilumab vs placebo groups. Least squares mean [95% CI] change from baseline at W24 in DSQ pain score was −10.1 [–12.2, –7.9] vs −4.4 [–6.8, –2.0] for dupilumab vs placebo in Part A (difference vs placebo: −5.7 [95% CI −8.67, –2.72]; *P*=0.0002), and −10.0 [–11.7, –8.1] vs −6.5 [–8.3, –4.6] in Part B (difference vs placebo: −3.5 [95% CI −5.90, −1.03]; *P*=0.0053).

**Conclusions:**

At W24, patients in the dupilumab 300 mg qw group experienced fewer days with dysphagia, fewer days with any action taken for dysphagia relief, and less pain associated with dysphagia vs placebo.

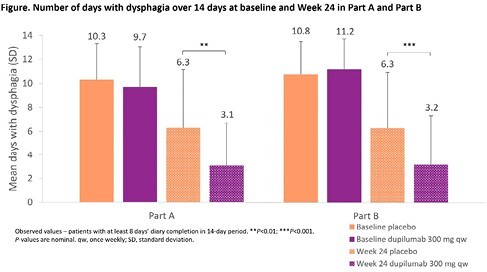

**Funding Agencies:**

Research sponsored by Sanofi and Regeneron Pharmaceuticals Inc.

